# Supplementary material for: Tumor suppressor Par-4 activates autophagy-dependent ferroptosis
Source: Commun Biol. 2024 Jun 17;7:732. doi: 10.1038/s42003-024-06430-z (PMC11183062; doi:10.1038/s42003-024-06430-z)
Supplement: Supplementary file 2 — Supplementary Information [file 42003_2024_6430_MOESM2_ESM.pdf]

**Table of Contents**

**Supplementary Figures**

Supplementary Figure 1. RSL3 or erastin induces non-apoptotic and non-necroptotic cell death

Supplementary Figure 2. Par-4 activation is essential for ferroptosis

Supplementary Figure 3. Par-4 regulates ferroptosis through autophagy

Supplementary Figure 4. Par-4 regulates ferroptosis by inducing ferritinophagy activation

Supplementary Figure 5. Par-4-dependent ROS accumulation is critical for autophagy mediates-ferroptosis

Supplementary Figure 6. Par-4 mediates the anticancer activity of RSL3 in vivo.

**Supplementary Tables**

Supplementary Table 1. Antibodies used in this study

Supplementary Table 2. Primers used for quantitative PCR

Supplementary Table 3. Sequences used for CRISPR/Cas9-mediated knockout of Par-4

Supplementary Table 4. Sequences used for shRNA-mediated knockdown of Atg-5 and Atg-7

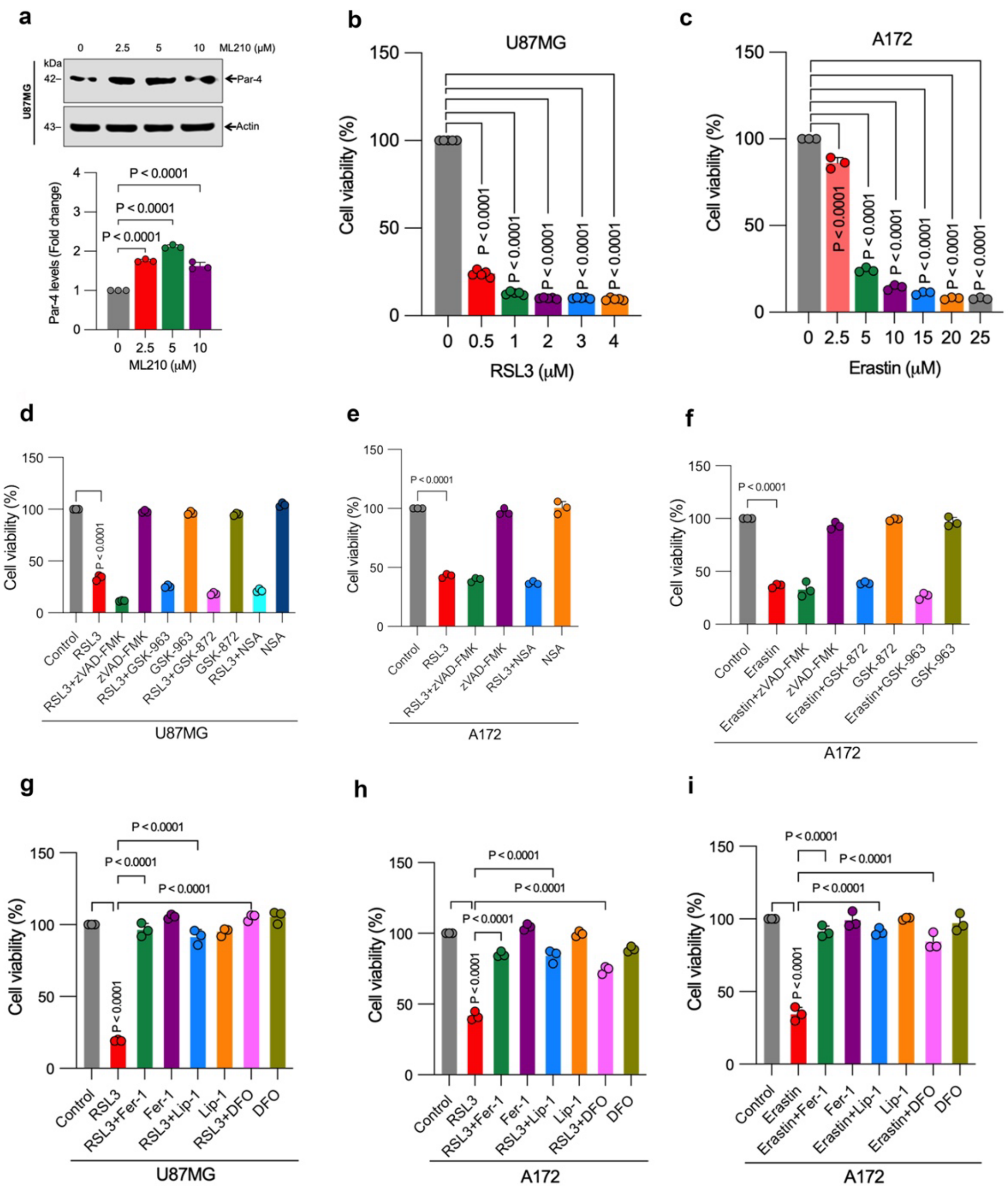
a
0 2.5 5 10 ML210 (μM)
kDa
42–
43–
U87MG
Par-4
Actin
Par-4 levels (Fold change)
P < 0.0001
P < 0.0001
P < 0.0001
ML210 (μM)
b
U87MG
Cell viability (%)
RSL3 (μM)
P < 0.0001
c
A172
Erastin (μM)
P < 0.0001
d
U87MG
Control
RSL3
RSL3+zVAD-FMK
zVAD-FMK
RSL3+GSK-963
GSK-963
RSL3+GSK-872
GSK-872
RSL3+NSA
NSA
e
A172
f
Erastin
Erastin+zVAD-FMK
Erastin+GSK-872
Erastin+GSK-963
g
RSL3+Fer-1
Fer-1
RSL3+Lip-1
Lip-1
RSL3+DFO
DFO
h
i
Erastin+Fer-1
Erastin+Lip-1
Erastin+DFO

**Supplementary Figure 1. RSL3 or erastin induces non-apoptotic and non-necroptotic cell death: a** U87MG cells were treated with the indicated concentration of ML210 for 3 h, and then Western blot analysis of Par-4 was determined. The relative density of protein bands were quantified and normalized to the actin of each group, and fold changes were presented in histograms from three independent experiments. Data shown are mean ± SD; n = 3 samples. Dose-responsive effect of RSL3 **b** and erastin **c** in glioma cells. U87MG and A172 cells were treated with the indicated concentration of RSL3 or erastin for 24 h. Cell viability was analyzed by MTT assay. Data shown are mean ± SD; n = 3 samples. **d** U87MG cells pre-treated with apoptosis inhibitor, z-VAD-FMK (50 μM), and necroptosis inhibitors such as GSK 963 (5 μM), GSK 872 (5 μM), and NSA (5 μM) for 1 h followed by RSL3 (2 μM) treatment for further 24 h. Cell viability was measured by using an MTT assay. Data shown are mean ± SD; n = 3 samples. **e** A172 cells pre-treated with z-VAD-FMK (50 μM) and NSA (5 μM) for 1 h were treated with RSL3 (2 μM) for 24 h. Cell viability was measured by using an MTT assay. Data shown are mean ± SD; n = 3 samples. **f** A172 cells pre-treated with z-VAD-FMK (50 μM), GSK 963 (5 μM), and GSK 872 (5 μM) for 1 h were treated with erastin (10 μM) for 24 h. Cell viability was measured by using an MTT assay. Data shown are mean ± SD; n = 3 samples. **g** U87MG and **h** A172 cells were treated with RSL3 (2 μM), and **i** A172 cells were treated with erastin (10 μM) for 24 h in the presence or absence of Fer-1 (5 μM), Lip-1 (1 μM), and DFO (100 μM). Cell viability was measured by using an MTT assay. Data shown are mean ± SD; n = 3 samples. Statistical significance (P values) was analyzed by one-way ANOVA using the Bonferroni post-hoc test.

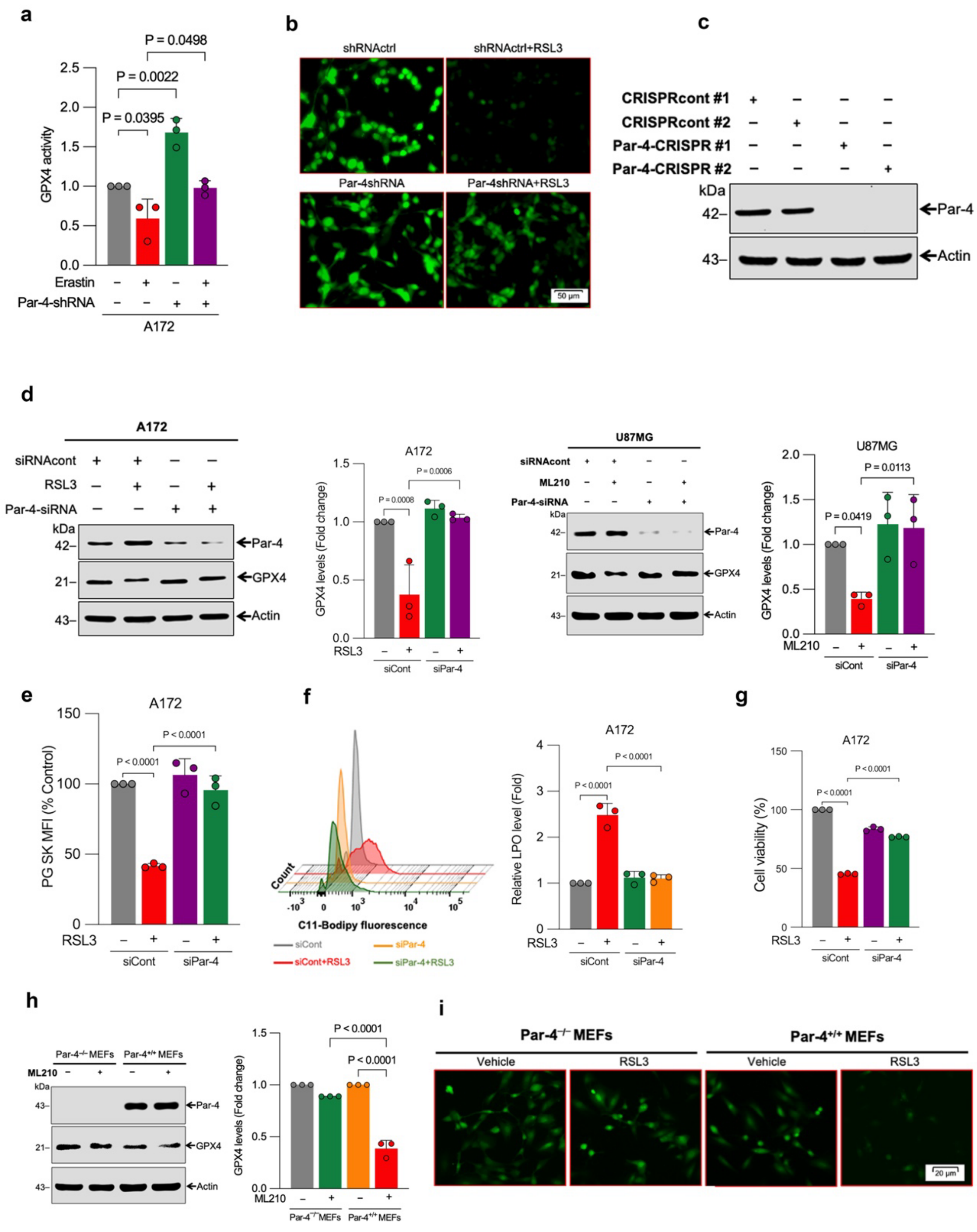

a
P = 0.0498
P = 0.0022
P = 0.0395
GPX4 activity
Erastin
Par-4-shRNA
A172
b
shRNActrl
shRNActrl+RSL3
Par-4shRNA
Par-4shRNA+RSL3
50 μm
c
CRISPRcont #1
CRISPRcont #2
Par-4-CRISPR #1
Par-4-CRISPR #2
kDa
Par-4
Actin
d
A172
siRNAcont
RSL3
Par-4-siRNA
GPX4
GPX4 levels (Fold change)
P = 0.0008
P = 0.0006
siCont
siPar-4
U87MG
ML210
P = 0.0419
P = 0.0113
e
PG SK MFI (% Control)
P < 0.0001
f
Count
C11-Bodipy fluorescence
siCont
siPar-4
siCont+RSL3
siPar-4+RSL3
Relative LPO level (Fold)
g
Cell viability (%)
h
Par-4−/− MEFs
Par-4+/+ MEFs
i
Vehicle
RSL3
20 μm

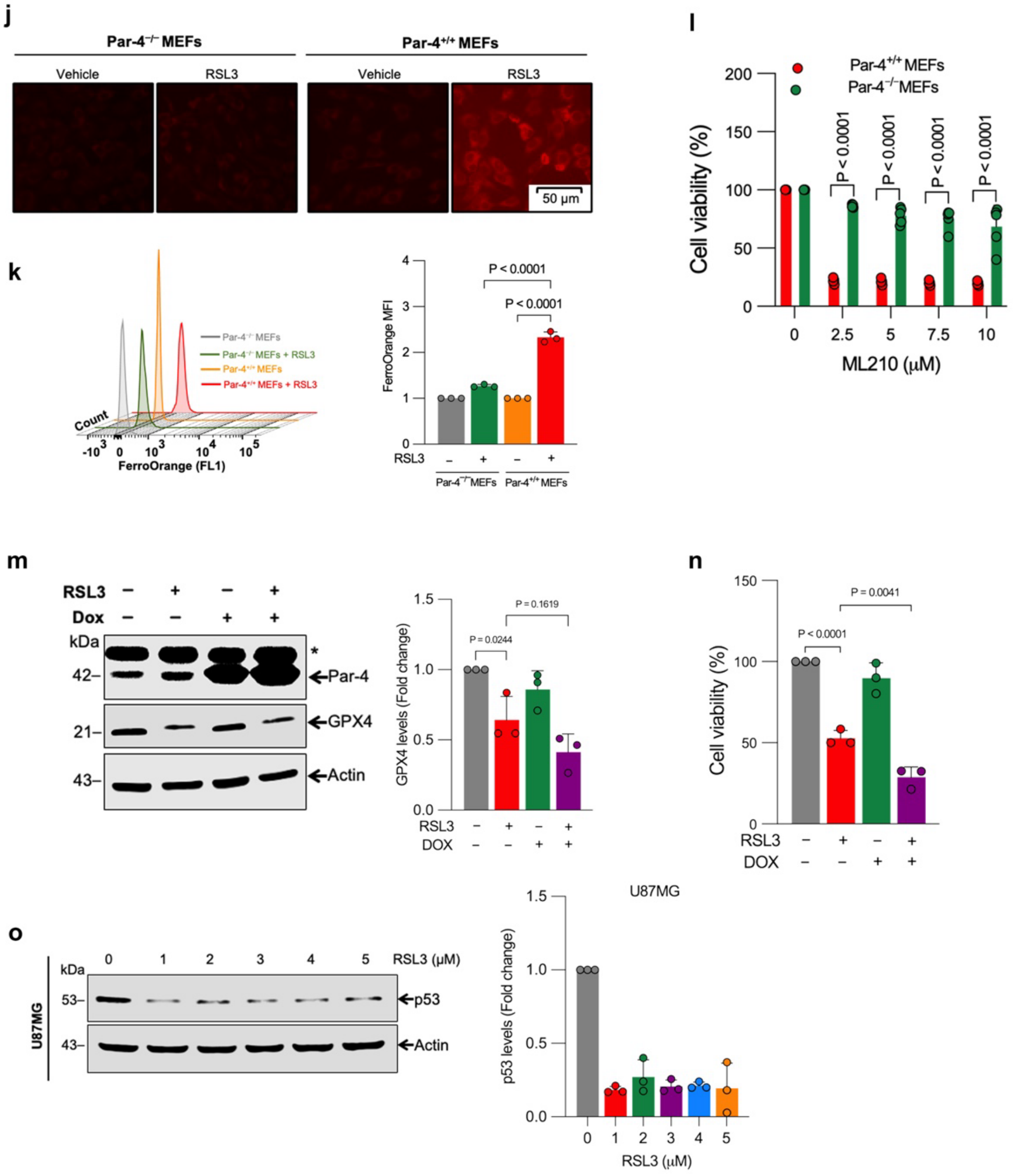
j
Par-4−/− MEFs
Par-4+/+ MEFs
Vehicle
RSL3
Vehicle
RSL3
50 µm
l
Par-4+/+ MEFs
Par-4−/− MEFs
Cell viability (%)
P < 0.0001
P < 0.0001
P < 0.0001
P < 0.0001
ML210 (µM)
k
Par-4−/− MEFs
Par-4−/− MEFs + RSL3
Par-4+/+ MEFs
Par-4+/+ MEFs + RSL3
Count
FerroOrange (FL1)
FerroOrange MFI
P < 0.0001
P < 0.0001
RSL3
Par-4−/− MEFs
Par-4+/+ MEFs
m
RSL3
Dox
kDa
Par-4
GPX4
Actin
GPX4 levels (Fold change)
P = 0.0244
P = 0.1619
DOX
n
Cell viability (%)
P < 0.0001
P = 0.0041
RSL3
DOX
o
U87MG
kDa
RSL3 (µM)
p53
Actin
p53 levels (Fold change)
RSL3 (µM)

**Supplementary Figure 2. Par-4 activation is essential for ferroptosis: a** A172 cells were stably transfected with Par-4-shRNA. After transfection, cells were treated with erastin (10 μM) for 24 h. Following the treatment, GPX4 activity was measured. Data shown are mean ± SD; n = 3 samples. **b** U87MG cells were stably transfected with Par-4-shRNA. After transfection, cells were treated with RSL3 (2 μM) for 3 h. Following the treatment, intracellular labile iron was determined using the fluorescent indicator PG SK by fluorescent microscope. **c** U87MG cells were stably transfected with the Par-4-CRISPR Cas9 system. After transfection, Par-4 knockout efficiency was validated by Western blot analysis. U87MG cells were transiently transfected with siPar-4. After transfection, cells were treated with ML210 (2.5 μM) for 3 h. Similarly, A172 cells were transiently transfected with siPar-4. After transfection, cells were treated with RSL3 (2 μM) for 3 h. Following the treatment, **d** Western blot analysis of Par-4 and GPX4 were performed. The relative density of protein bands were quantified and normalized to the actin of each group, and fold changes were presented in histograms from three independent experiments. **e** Intracellular labile iron was determined using flow cytometry. The bar graph showing labile iron level was expressed as a percentage of the control. **f** Lipid peroxidation was detected by flow cytometry. Bar graphs showing the relative lipid peroxidation levels and **g** Cell viability were performed. Data shown are mean ± SD; n = 3 samples. Par-$4^{+/+}$ and Par-$4^{-/-}$ MEFs were treated with ML210 (2.5 μM) for 3 h. Following the treatment, **h** Western blot analysis of indicated proteins were detected. The relative density of protein bands were quantified and normalized to the actin of each group, and fold changes were presented in histograms from three independent experiments. Data shown are mean ± SD; n = 3 samples. **i** Par-$4^{+/+}$ and Par-$4^{-/-}$ MEFs were treated with RSL3 (2 μM) for 3 h. Following the treatment, intracellular labile iron was determined using the fluorescent indicator PG SK by fluorescent microscope. Par-$4^{+/+}$ MEFs and Par-$4^{-/-}$ MEFs were also treated with RSL3

(2 μM) for 3 h. After the treatment, the cells were subjected to FerroOrange staining to evaluate intracellular LIP **j**. Red fluorescence signals were captured and visualized through a fluorescent microscope using constant fluorescence parameters described in the methods section: scale bar, 50 μm. Subsequently, median fluorescence intensity (MFI) was quantified by flow cytometry analysis. The bar graph shows relative levels of LIP by FerroOrange staining in the indicated cells **k**. Data shown are mean ± SD; n = 3 samples. Par-4$^{+/+}$ and Par-4$^{-/-}$ MEFs were treated with ML210 (2.5 μM) for 3 h. Following the treatment, **l** Cell viability were assessed. Data shown are mean ± SD; n = 3 samples. Dox-ON-Par-4-U87MG cells were treated with Dox for 12 h, followed by RSL3 (2 μM) for 3 h. After the treatment, **m** Western blot analysis of indicated proteins were detected. The relative density of protein bands were quantified and normalized to the actin of each group, and fold changes were presented in histograms from three independent experiments, and **n** Cell viability were assessed. Data shown are mean ± SD; n = 3 samples. **o** U87MG cells were treated with the indicated concentration of RSL3 for 24 h, and then Western blot analysis of p53 was determined. The relative density of protein bands were quantified and normalized to the actin of each group, and fold changes were presented in histograms from three independent experiments. *non-specific band. Data shown are mean ± SD; n = 3 samples. Statistical significance (P values) was analyzed by one-way ANOVA using the Bonferroni post-hoc test.

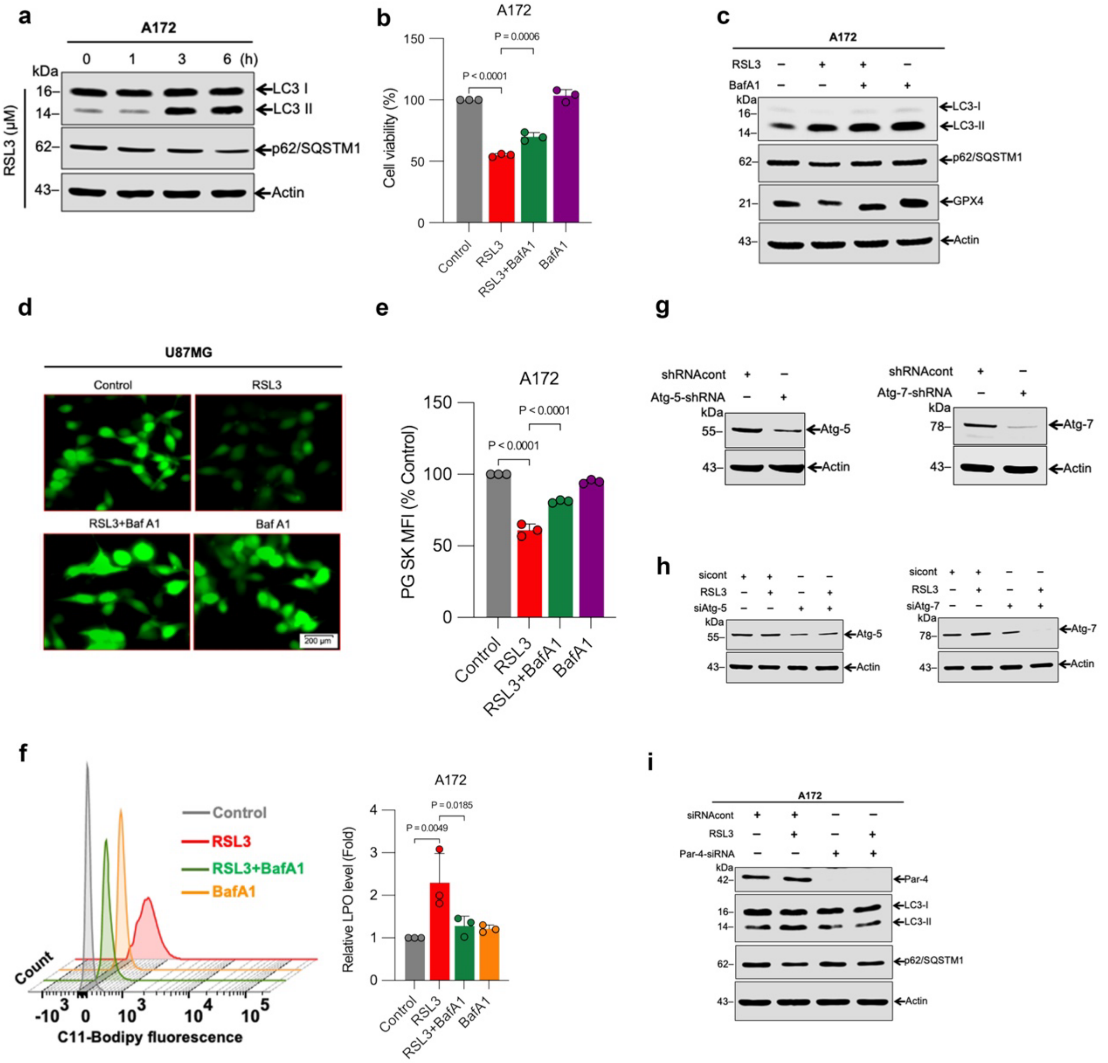

a
A172
0 1 3 6 (h)
kDa
RSL3 (μM)
16–
14–
62–
43–
LC3 I
LC3 II
p62/SQSTM1
Actin
b
A172
Cell viability (%)
P < 0.0001
P = 0.0006
Control
RSL3
RSL3+BafA1
BafA1
c
A172
RSL3
BafA1
LC3-I
LC3-II
p62/SQSTM1
GPX4
Actin
d
U87MG
Control
RSL3
RSL3+Baf A1
Baf A1
200 μm
e
A172
PG SK MFI (% Control)
P < 0.0001
P < 0.0001
f
Control
RSL3
RSL3+BafA1
BafA1
Count
C11-Bodipy fluorescence
A172
Relative LPO level (Fold)
P = 0.0049
P = 0.0185
g
shRNAcont
Atg-5-shRNA
Atg-7-shRNA
Atg-5
Atg-7
h
sicont
siAtg-5
siAtg-7
i
siRNAcont
Par-4-siRNA
Par-4

j

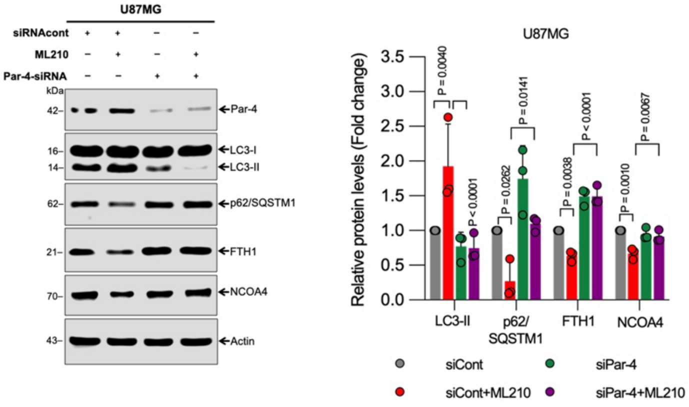
U87MG
siRNAcont
ML210
Par-4-siRNA
kDa
42–
16–
14–
62–
21–
70–
43–
Par-4
LC3-I
LC3-II
p62/SQSTM1
FTH1
NCOA4
Actin
U87MG
Relative protein levels (Fold change)
3.5
3.0
2.5
2.0
1.5
1.0
0.5
0.0
P = 0.0040
P < 0.0001
P = 0.0262
P = 0.0141
P = 0.0038
P < 0.0001
P = 0.0010
P = 0.0067
LC3-II
p62/ SQSTM1
FTH1
NCOA4
siCont
siPar-4
siCont+ML210
siPar-4+ML210

k

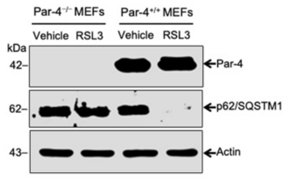
Par-4$^{-/-}$ MEFs
Par-4$^{+/+}$ MEFs
Vehicle
RSL3
Vehicle
RSL3
kDa
42–
62–
43–
Par-4
p62/SQSTM1
Actin

l

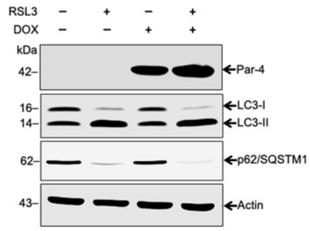
RSL3
DOX
kDa
42–
16–
14–
62–
43–
Par-4
LC3-I
LC3-II
p62/SQSTM1
Actin

m

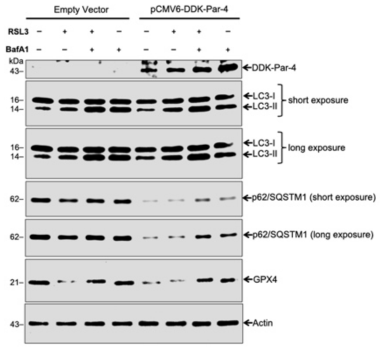
Empty Vector
pCMV6-DDK-Par-4
RSL3
BafA1
kDa
43–
16–
14–
62–
21–
DDK-Par-4
LC3-I
LC3-II
short exposure
long exposure
p62/SQSTM1 (short exposure)
p62/SQSTM1 (long exposure)
GPX4
Actin

**Supplementary Figure 3. Par-4 regulates ferroptosis through autophagy. a** A172 cells were treated with RSL3 (2 μM) for the indicated time period. Following the treatment, a Western blot analysis of indicated proteins were carried out. Actin was used as a loading control. The blots shown are representative blots from three independent experiments. **b** A172 cells were treated with RSL3 (2 μM) for 24 h in the presence or absence of BafA1 (250 nM). Cell viability was measured by using an MTT assay. Data shown are mean ± SD; n = 3 samples. A172 cells were treated with RSL3 (2 μM) for 3 h in the presence or absence of BafA1 (250 nM). Following treatment, **c** Western blot analysis of indicated proteins were carried out. Actin was used as a loading control. The blots shown are representative blots from three independent experiments. **d** U87MG cells were treated with RSL3 (2 μM) for 3 h in the presence or absence of BafA1 (250 nM). Following the treatment, intracellular labile iron were determined using the fluorescent indicator PG SK by fluorescent microscope. A172 cells were treated with RSL3 (2 μM) for 24 h in the presence or absence of BafA1 (250 nM). Following the treatment, **e** Intracellular labile iron was determined using flow cytometry. The bar graph showing labile iron level was expressed as a percentage of the control, and **f** Lipid peroxidation was detected by flow cytometry. Bar graph showing the relative levels of lipid peroxidation. Data shown are mean ± SD; n = 3 samples. **g** U87MG cells were stably transfected with Atg-5 and Atg-7 by the shRNA. After transfection, Atg-5 and Atg-7 knockdown efficiency was validated by Western blot analysis. **h** U87MG cells were transiently transfected with siAtg-5 and siAtg-7, then exposed to 2 μM RSL3 for 3 h. After transfection, Atg-5 and Atg-7 knockdown efficiency was validated by Western blot analysis. **i** A172 cells were transiently transfected with siPar-4 followed by exposure to 2 μM RSL3 for 3 h. **j** Similarly, U87MG cells were transiently transfected with siPar-4. After transfection, cells were treated with ML210 (2.5 μM) for 3 h. Following the treatment, a Western blot analysis of indicated proteins

were carried out. The relative density of protein bands were quantified and normalized to the actin of each group, and fold changes were presented in histograms from three independent experiments. Data shown are mean ± SD; n = 3 samples. **k** Par-4$^{+/+}$ and Par-4$^{-/-}$ MEFs were treated with RSL3 (2 µM) for 3 h. Following the treatment, a Western blot analysis of indicated proteins were carried out. Actin was used as a loading control. The blots shown are representative blots from three independent experiments. **l** Dox-ON-Par-4-U87MG cells were treated with Dox for 12 h, followed by RSL3 (2 µM) for 3 h. After the treatment, a Western blot analysis of indicated proteins were detected. Actin was used as a loading control. The blots shown are representative blots from three independent experiments. **m** A172 cells were transiently transfected with either 1 µg of the empty vector or the Par-4 endogenous human pCMV6-DDK vector. After the transfection, cells were treated with RSL3 2 µM for 3 h in the presence or absence of 250 nM Bafilomycin A1 (BafA1). Whole-cell lysates were immunoblotted to detect DDK-Par-4, LC3, p62, and GPX4. Actin was used as a loading control. Statistical significance (P values) was analyzed by one-way ANOVA using the Bonferroni post-hoc test.

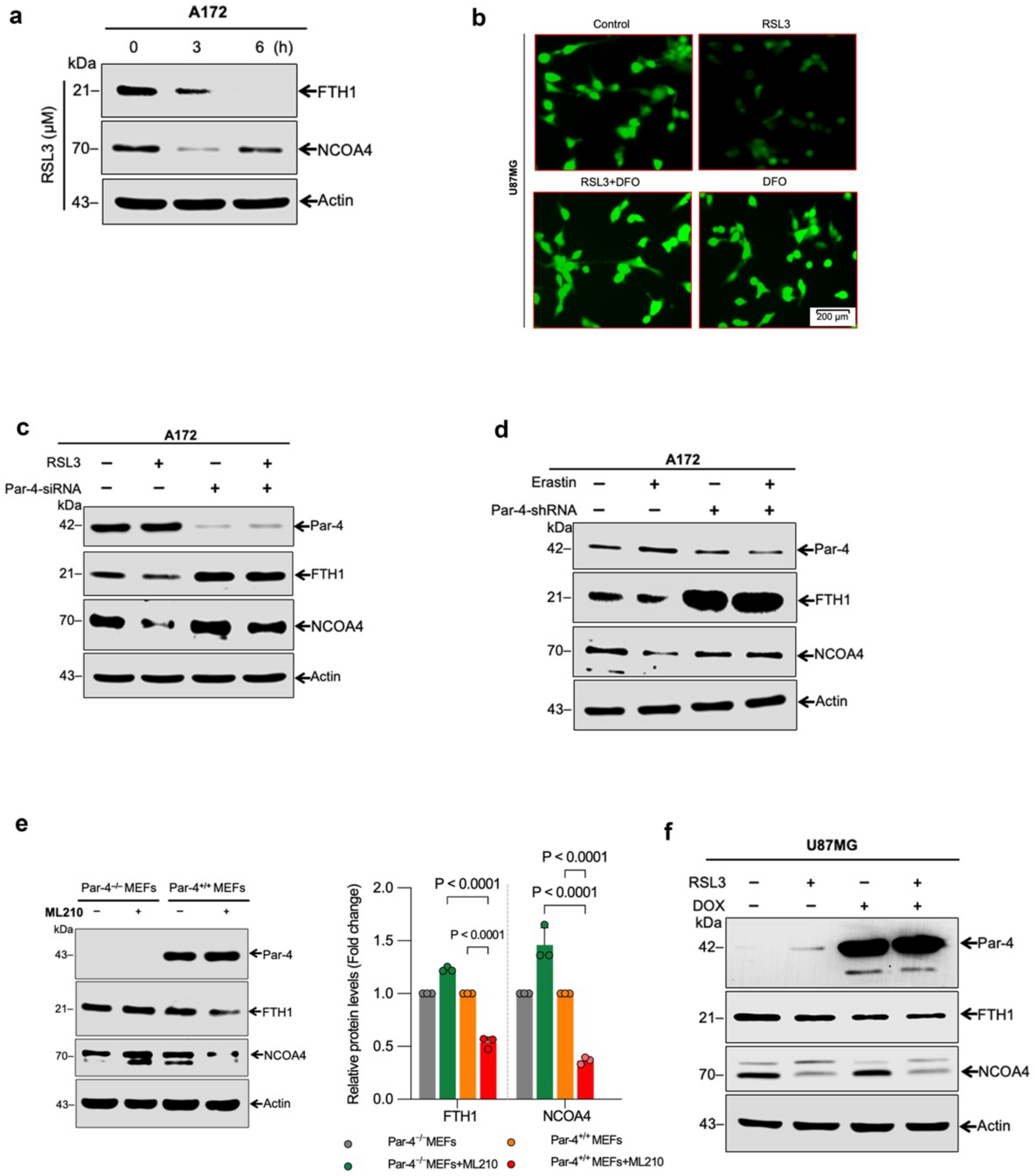

a
A172
0
3
6 (h)
kDa
RSL3 (μM)
21–
70–
43–
FTH1
NCOA4
Actin
b
U87MG
Control
RSL3
RSL3+DFO
DFO
200 μm
c
A172
RSL3
Par-4-siRNA
kDa
42–
21–
70–
43–
Par-4
FTH1
NCOA4
Actin
d
A172
Erastin
Par-4-shRNA
kDa
42–
21–
70–
43–
Par-4
FTH1
NCOA4
Actin
e
Par-4−/− MEFs
Par-4+/+ MEFs
ML210
kDa
43–
21–
70–
43–
Par-4
FTH1
NCOA4
Actin
Relative protein levels (Fold change)
2.0
1.5
1.0
0.5
0.0
P < 0.0001
P < 0.0001
P < 0.0001
P < 0.0001
FTH1
NCOA4
Par-4−/− MEFs
Par-4−/− MEFs+ML210
Par-4+/+ MEFs
Par-4+/+ MEFs+ML210
f
U87MG
RSL3
DOX
kDa
42–
21–
70–
43–
Par-4
FTH1
NCOA4
Actin

**Supplementary Figure 4. Par-4 regulates ferroptosis by inducing ferritinophagy activation.** **a** A172 cells were treated with RSL3 (2 μM) for the indicated time period, and then Western blot analysis of FTH1 and NCOA4 were performed. Actin was used as a loading control. The blots shown are representative blots from three independent experiments. U87MG cells were treated with RSL3 (2 μM) for 3 h in the presence or absence of DFO (100 μM). Following the treatment, **b** Intracellular labile iron was determined using the fluorescent indicator PG SK by fluorescent microscope. A172 cells were stably transfected with Par-4-shRNA. After transfection, cells were treated with **c** RSL3 (2 μM) for 3 h and **d** erastin (10 μM) for 24 h, and then a Western blot analysis of indicated proteins were carried out. Actin was used as a loading control. The blots shown are representative blots from three independent experiments. Par-$4^{+/+}$ and Par-$4^{-/-}$ MEFs were treated with ML210 (2.5 μM) for 3 h. Following the treatment, **e** Western blot analysis of indicated proteins were carried out. The relative density of protein bands were quantified and normalized to the actin of each group, and fold changes were presented in histograms from three independent experiments. Data shown are mean ± SD; n = 3 samples. **f** Dox-ON-Par-4-U87MG cells were treated with Dox for 12 h, followed by RSL3 (2 μM) for 3 h. After the treatment, a Western blot analysis of indicated proteins were detected. Actin was used as a loading control. The blots shown are representative blots from three independent experiments. Data shown are mean ± SD; n = 3 samples. Statistical significance (P values) was analyzed by one-way ANOVA using the Bonferroni post-hoc test.

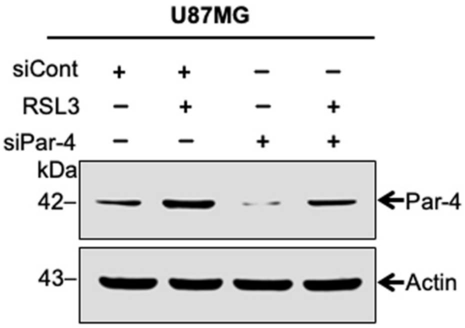
U87MG
siCont
RSL3
siPar-4
kDa
42–
Par-4
43–
Actin

**Supplementary Figure 5. Par-4-dependent ROS accumulation is critical for autophagy mediates-ferroptosis.** U87MG cells were transiently transfected with siPar-4, followed by exposure to 2 μM RSL3 for 3 h. Following the treatment, a Western blot analysis of Par-4 was performed, and the efficiency of Par-4 knockdown was examined. Actin was used as a loading control. The blots shown are representative blots from three independent experiments.

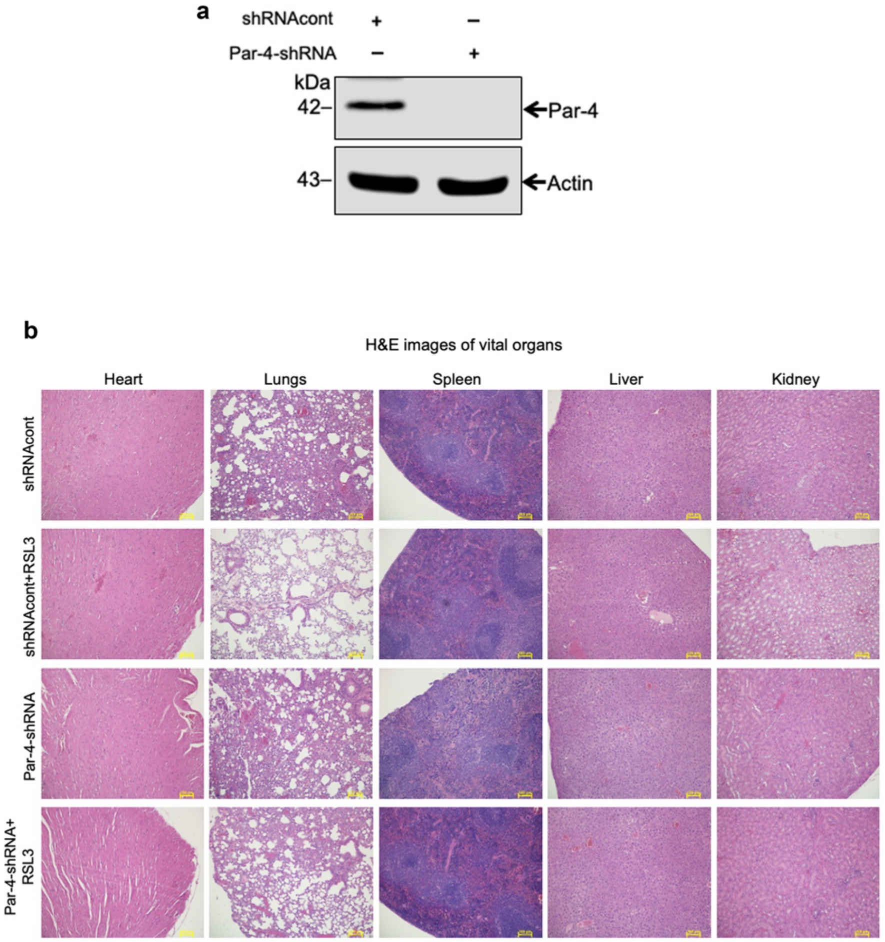

a
shRNAcont + −
Par-4-shRNA − +
kDa
42–
Par-4
43–
Actin
b
H&E images of vital organs
Heart
Lungs
Spleen
Liver
Kidney
shRNAcont
shRNAcont+RSL3
Par-4-shRNA
Par-4-shRNA+ RSL3

**Supplementary Figure 6. Par-4 mediates the anticancer activity of RSL3 in vivo. a** Western blot analysis confirmed the efficiency of Par-4 knockdown U87MG after the end of the treatment in isolated tumor tissues. Actin was used as a loading control. The blots shown are representative blots from three independent experiments. **b** Histologically examined vital organs following treatment with RSL3 in control shRNA and Par-4 stable knockdown U87MG tumor xenografts. Hematoxylin and eosin (H&E) staining of heart, lung, spleen, liver, and kidney sections from shRNA control and Par-4 knockdown U87MG tumor tumor-bearing mice after 21 days of treatment with saline or RSL3 (dosage of 4.4 mg/kg in saline, administered every 2 days, for a total of 10 doses). The images shown represent tissue sections from four mice per treatment group: scale bar, 100 μm.

**Supplementary Table 1. Antibodies used in this study**

| Antibodies | Dilution | Source | Identifier |
|---|---|---|---|
| Par-4 | 1:1000 | Santa Cruz Biotechnology | #Sc-130078 |
| Actin | 1:1000 | Santa Cruz Biotechnology | #Sc-47778 |
| p62/SQSTM1 | 1:2000 | Santa Cruz Biotechnology | #Sc-28359 |
| FTH1(C-terminal) | 1:500 | Cell Signaling Technology | #4393S |
| FTH1 (N-terminal) | 1:500 | Cell Signaling Technology | #3998 |
| LC3B (D11) XP | 1:1000 | Cell Signaling Technology | #3868 |
| GPX4 | 1:1000 | Abcam | #ab125066 |
| NCOA4 | 1:1000 | Abcam | #ab86707 |
| DDK | 1:1000 | Origene | #TA50011-1 |
| LC3B | 1:1000 | Sigma-Aldrich | #L7543 |
| Peroxidase affinipure goat anti-mouse IgG | 1:1000 | Jackson Immuno Research | #115-035-003 |
| Peroxidase affinipure goat anti-rabbit IgG | 1:1000 | Jackson Immuno Research | #111-035-003 |

**Supplementary Table 2. Primers used for quantitative PCR**

| Gene | Primer | Sequence | Source | Identifier |
|---|---|---|---|---|
| Par-4/PAWR | Forward | 5′-GCCGCAGAGTGATTAGATGAG-3′ | IDT | This paper |
| | Reverse | 5′-GCAGATAGGAACTGCCTGGAT-3′ | IDT | This paper |
| GAPDH | Forward | 5′-GTCAGTGGTGGACCTGACCT-3’ | IDT | This paper |
| | Reverse | 5′-TGAGCTTGACAAAGTGGTCG-3′ | IDT | This paper |

**Supplementary Table 3. Sequences used for CRISPR/Cas9-mediated knockout of Par-4**

| Gene | Sequence | Source | Identifier |
|---|---|---|---|
| hPAWR | AGTTCACGCCCCCGGGACCG | Vector Builder (sgRNA#281) | This paper |
| hPAWR | ACCGCCGCCCCAGCGTGACG | Vector Builder (sgRNA#312) | This paper |

**Supplementary Table 4. Sequences used for shRNA-mediated knockdown of Atg-5 and Atg-7**

| Gene | Sequence | Source | Identifier |
|---|---|---|---|
| Atg-5-shRNA-1 | GTGCTTCGAGATGTGTGGTTTGGACGAAT | OriGene | TL314610 |
| Atg-5-shRNA-2 | TCAGCTCTTCCTTGGAACATCACAGTACA | OriGene | TL314610 |
| Atg-7-shRNA-1 | TGCCAGCTCGCTTAACATTGGAGTTCAGT | OriGene | TL314609 |
| Atg-7-shRNA-2 | CTTGGCTGCTACTTCTGCAATGATGTGGT | OriGene | TL314609 |
| shRNACont | GCACTACCAGAGCTAACTCAGATAGTACT | OriGene | TR30021 |
