# Supplementary material for: Tumor suppressor Par-4 activates autophagy-dependent ferroptosis
Source: Commun Biol. 2024 Jun 17;7:732. doi: 10.1038/s42003-024-06430-z (PMC11183062; doi:10.1038/s42003-024-06430-z)
Supplement: Supplementary file 3 — Description of Additional Supplementary Files [file 42003_2024_6430_MOESM3_ESM.pdf]

# Description of Additional Supplementary Files

File name: Supplementary Data 1

Description: This Excel file contains the RNA sequencing results.

File name: Supplementary Data 2

Description: Numerical source data for Figures 1- 6 and Supplementary Figures 1-6 behind the graphs in the paper.
