# Supplementary material for: Tumor suppressor Par-4 activates autophagy-dependent ferroptosis
Source: Commun Biol. 2024 Jun 17;7:732. doi: 10.1038/s42003-024-06430-z (PMC11183062; doi:10.1038/s42003-024-06430-z)
Supplement: Supplementary file 6 — Reporting summary [file 42003_2024_6430_MOESM6_ESM.pdf]

nature portfolio

Corresponding author(s): Sehamuddin Galadari

Last updated by author(s): May 20, 2024

Reporting Summary

Nature Portfolio wishes to improve the reproducibility of the work that we publish. This form provides structure for consistency and transparency in reporting. For further information on Nature Portfolio policies, see our Editorial Policies and the Editorial Policy Checklist.

Statistics

For all statistical analyses, confirm that the following items are present in the figure legend, table legend, main text, or Methods section.

n/a	Confirmed	
☐	☒	The exact sample size (*n*) for each experimental group/condition, given as a discrete number and unit of measurement
☐	☒	A statement on whether measurements were taken from distinct samples or whether the same sample was measured repeatedly
☐	☒	The statistical test(s) used AND whether they are one- or two-sided<br>*Only common tests should be described solely by name; describe more complex techniques in the Methods section.*
☒	☐	A description of all covariates tested
☒	☐	A description of any assumptions or corrections, such as tests of normality and adjustment for multiple comparisons
☐	☒	A full description of the statistical parameters including central tendency (e.g. means) or other basic estimates (e.g. regression coefficient) AND variation (e.g. standard deviation) or associated estimates of uncertainty (e.g. confidence intervals)
☐	☒	For null hypothesis testing, the test statistic (e.g. *F*, *t*, *r*) with confidence intervals, effect sizes, degrees of freedom and *P* value noted<br>*Give P values as exact values whenever suitable.*
☒	☐	For Bayesian analysis, information on the choice of priors and Markov chain Monte Carlo settings
☒	☐	For hierarchical and complex designs, identification of the appropriate level for tests and full reporting of outcomes
☒	☐	Estimates of effect sizes (e.g. Cohen's *d*, Pearson's *r*), indicating how they were calculated

Our web collection on statistics for biologists contains articles on many of the points above.

Software and code

Policy information about availability of computer code

Data collection	BD FACSAriaIII and Canto II was used to collect flow cytometry data. PerkinElmer EnSpire multimode plate reader was used to collect ROS and GPX4 level.
Data analysis	GraphPad Prism 10.1.1 was used to collect and analyze data. Q-PCR analysis was performed using Applied Biosystems StepOnePlus software V.2.3. FlowJo V.10.1 software was used for flow data analysis. Western blots were analyzed using Image Studio Lite 5.2.5 software (LI-COR Biosciences). Microsoft Excel was used for statistical analysis.

For manuscripts utilizing custom algorithms or software that are central to the research but not yet described in published literature, software must be made available to editors and reviewers. We strongly encourage code deposition in a community repository (e.g. GitHub). See the Nature Portfolio guidelines for submitting code & software for further information.

Data

Policy information about availability of data

All manuscripts must include a data availability statement. This statement should provide the following information, where applicable:

- Accession codes, unique identifiers, or web links for publicly available datasets
- A description of any restrictions on data availability
- For clinical datasets or third party data, please ensure that the statement adheres to our policy

All the data supporting of this study are available within the article and its supplementary information files.

March 2021

Human research participants

Policy information about studies involving human research participants and Sex and Gender in Research.

Reporting on sex and gender	No human research involved.
Population characteristics	No human research involved.
Recruitment	No human research involved.
Ethics oversight	No human research involved.

Note that full information on the approval of the study protocol must also be provided in the manuscript.

Field-specific reporting

Please select the one below that is the best fit for your research. If you are not sure, read the appropriate sections before making your selection.

☒ Life sciences ☐ Behavioural & social sciences ☐ Ecological, evolutionary & environmental sciences

For a reference copy of the document with all sections, see nature.com/documents/nr-reporting-summary-flat.pdf

Life sciences study design

All studies must disclose on these points even when the disclosure is negative.

Sample size	Power calculation was used to select sample sizes from the NYU Abu Dhabi Institutional Animal Care and Use Committee (NYUAD-IACUC) Protocol (Protocol No. 21-0005)
Data exclusions	No data was excluded.
Replication	Replicates in all assays were confirmatory and the extent described within the text and shown in the main figures.
Randomization	For the tumor inhibition studies, tumor bearing mice were randomized into the treatment groups.
Blinding	For RNA-seq experiments, cell types were known when prepare the RNA samples or start to treat cell at the beginning of experiments. Data measurement for RNA-seq, were blinded to different person who processed assay or analysis at the time. For in vivo studies, investigators were blinded for some parts of the experiments (animal work supervision, design and data analysis).

Reporting for specific materials, systems and methods

We require information from authors about some types of materials, experimental systems and methods used in many studies. Here, indicate whether each material, system or method listed is relevant to your study. If you are not sure if a list item applies to your research, read the appropriate section before selecting a response.

Materials & experimental systems

n/a	Involved in the study	
☐	☒	Antibodies
☐	☒	Eukaryotic cell lines
☒	☐	Palaeontology and archaeology
☐	☒	Animals and other organisms
☒	☐	Clinical data
☒	☐	Dual use research of concern

Methods

n/a	Involved in the study	
☒	☐	ChIP-seq
☐	☒	Flow cytometry
☒	☐	MRI-based neuroimaging

Antibodies

Antibodies used	Mouse anti-PAR4 monoclonal antibody SCBT Sc-130078 (3G9H7), WB 1:1000<br>Mouse anti-Actin monoclonal antibody, SCBT Sc-47778, WB 1:1000<br>Mouse anti-SQSTM1 (D-3) monoclonal antibody, SCBT Sc-28359, WB 1:2000<br>Rabbit anti-FTH1 polyclonal antibody, CST 3998, WB 1:500<br>Rabbit anti-NCOA4 polyclonal antibody, Abcam ab86707, WB 1:1000

	Peroxidase-AffiniPure goat anti-mouse IgG (H + L), Jackson 115-035-003, WB 1:1000<br>Rabbit anti-LC3B (D11) XP, CST 3868, WB 1:1000<br>Anti-DDK (FLAG) monoclonal antibody, Origene TA50011-1, WB 1:500<br>Rabbit anti-Glutathione peroxidase 4 monoclonal antibody, Abcam ab125066, WB 1:1000<br>Peroxidase-AffiniPure Goat Anti-Rabbit IgG (H+L), Jackson 111-035-003, WB 1:1000
Validation	All antibodies used in our study have been validated and detailed information could be obtained the website from manufactures as listed below:<br>Par-4: https://www.scbt.com/p/par4-antibody-3g9h7<br>Actin: https://www.scbt.com/p/beta-actin-antibody-c4?requestFrom=search<br>SQSTM1/p62: https://www.scbt.com/p/sqstm1-antibody-d-3?requestFrom=search<br>FTH1: https://www.cellsignal.com/products/primary-antibodies/fth1-antibody/3998<br>NCOA4: https://www.abcam.com/products/primary-antibodies/ncoa4-antibody-ab86707.html<br>Peroxidase affinipure goat anti-mouse IgG: https://www.jacksonimmuno.com/catalog/products/115-035-003<br>LC3 (XP): https://www.cellsignal.com/product/productDetail.jsp?productId=3868<br>DDK: https://www.origene.com/catalog/antibodies/tag-antibodies/ta50011-1/clone-oti4c5-anti-ddk-flag-monoclonal-antibody<br>GPX4: https://www.abcam.com/products/primary-antibodies/glutathione-peroxidase-4-antibody-epncir144-ab125066.html<br>Peroxidase affinipure goat anti-rabbit IgG: https://www.jacksonimmuno.com/catalog/products/111-035-003

Eukaryotic cell lines

Policy information about cell lines and Sex and Gender in Research

Cell line source(s)	Human glioblastoma cell line U87MG was purchased from American Type Culture Collection (ATCC, USA) and A172 cell line was purchased from European Collection of Authenticated Cell Cultures (ECACC, UK).
Authentication	Cell lines were authenticated by Charles River Laboratories
Mycoplasma contamination	Testing for mycolplasma contamination was done by Charles River Laboratories
Commonly misidentified lines (See ICLAC register)	No ICLAC cell line was used in this study

Animals and other research organisms

Policy information about studies involving animals; ARRIVE guidelines recommended for reporting animal research, and Sex and Gender in Research

Laboratory animals	Xenograft Studies: Female Balb/c nude mice (The Jackson Laboratory, Bar Harbor, ME) , 6-8 weeks old.
Wild animals	No wild animals involved in this study
Reporting on sex	Female mice were used in this study
Field-collected samples	No filed-collected samples were used in the study.
Ethics oversight	All animal experiments were approved by the NYU Abu Dhabi Institutional Animal Care and Use Committee (NYUAD-IACUC; Protocol No: 21-0005), and performed in accordance with the Guide for Care and Use of Laboratory Animals.

Note that full information on the approval of the study protocol must also be provided in the manuscript.

Flow Cytometry

Plots

Confirm that:

- [x] The axis labels state the marker and fluorochrome used (e.g. CD4-FITC).
- [x] The axis scales are clearly visible. Include numbers along axes only for bottom left plot of group (a 'group' is an analysis of identical markers).
- [x] All plots are contour plots with outliers or pseudocolor plots.
- [x] A numerical value for number of cells or percentage (with statistics) is provided.

Methodology

Sample preparation	Sample preparation details provided in the methods section and were based upon previously published report or manufactures protocol.
Instrument	BD FACSAriaIII and Canto II Flow cytometer

Software	BD FACSDiva software for data collection and FlowJo V.10.1 for data analysis
Cell population abundance	Samples were filtered prior to experimental using standard operating procedure. A minimum of 10,000 post staining cells were analyzed. for each condition. BoDIPY-C11 and PGSK or FerroOrange signal was confirmed on a fluorescence microscope.
Gating strategy	10,000 cells/sample (3 biological replicates), gated on live cells by forward/side scatter and using both FITC and PE-Texas Red filters.

☒ Tick this box to confirm that a figure exemplifying the gating strategy is provided in the Supplementary Information.
